# Case report: early aortic valve degeneration associated with interstitial deletion of chromosome 2–46,XX,del (2)(q31.3; q32.2)

**DOI:** 10.1186/s13019-020-01104-3

**Published:** 2020-04-21

**Authors:** Raymond Pfister, Clara Knowles, Matthias Kirsch

**Affiliations:** Department of Cardiovascular Surgery, Lausanne University Hospital (CHUV), CH-1011 Lausanne, Switzerland

**Keywords:** Genetic, Microdeletion, Valvular degeneration, Aortic valve insufficiency, Fibromyxoid degeneration

## Abstract

**Background:**

Interstitial deletions within the long arm of chromosome 2, involving the 2q31q33 region, are rare but are known to be associated with delays in development, behavioral problems, facial dysmorphism and various hand/foot anomalies.

**Case presentation:**

Here, we describe a case with an interstitial 2q31.3.q32.2 deletion, presenting the previously described phenotype, exhibiting fibromyxoid degeneration of the aortic valve in addition to previously described clinical features.

**Conclusion:**

Interstitial deletion in chromosome 2q31.2q32.3 might be associated to a fibromyxoid degeneration of valvular leaflets generating regurgitation. Patients diagnosed with this mutation may require investigation to rule out a valvular disease.

## Introduction

Interstitial deletions within the long arm of chromosome 2 involving the 2q31q33 region are rare, but their phenotypic features have been well characterised such as those of ‘2q31.2q32.3 deletion syndrome’ [1] which includes developmental delay, behavioral problems, facial dysmorphism and various hand/foot anomalies. Some cardiovascular anomalies have been described in association with mutations of other loci of chromosome 2 [2, 3], but this microdeletion has never before been associated with early aortic valve degeneration.

## Case report

The proband was a female born prematurely at 37 weeks due to a premature rupture of the membranes, with no acute fetal distress. She had a low birth weight of 2.68 kg with a post-natal Apgar score of 9–10-10. All post-natal examinations were normal except for an abnormal Barlow’s maneuver of the left lower limb. Throughout childhood, she remained below the 3rd and 10th percentiles for height and weight respectively and since her first year of life, she has had severe anemia with no attributable cause.

Alimentary issues began at birth and have persisted into adulthood. Factors at play were a buccal and peri-buccal hypersensitivity, an easily provoked emetic reflex and gastroesophageal reflux. Radiology in infancy also showed an organo-axial volvulus and a posterior projection of the antro-pyloro-bulbar region of the stomach.

A moderate global psychomotor delay and hetero-aggressive behaviour, documented since early childhood, have also persisted into adulthood. Outside of these non-epileptic episodes of aggression, she has a cheerful and playful disposition. Her musculature is globally hypotonic, she has flat feet bilaterally and intermittent strabismus of the left eye.

Aged 20 at the time of this report, the patient exhibits a high set forehead, a rolled-up nose and a triangular bottom half of the face. She also has small tonsils, an absent uvula, a small and anteriorly displaced soft palate as well as left septal deviation with keratoconus of both eyes. A severe dorso-lumbar scoliosis developed in her teenage years and she now has bone demineralisation with a documented estrogen deficit and abnormally high levels of testosterone. Menarche never began, but now taking an oral contraceptive pill, she experiences painful menstruation.

This patient is of particular interest to us because, although she exhibits some previously documented signs of an interstitial chromosome 2 deletion, at age 20 she developed a severe, but symptomless, aortic insufficiency (regurgitant volume of 95 ml, regurgitant orifice of 40mm^2^) which has not been observed before.

**Fig. 1:**
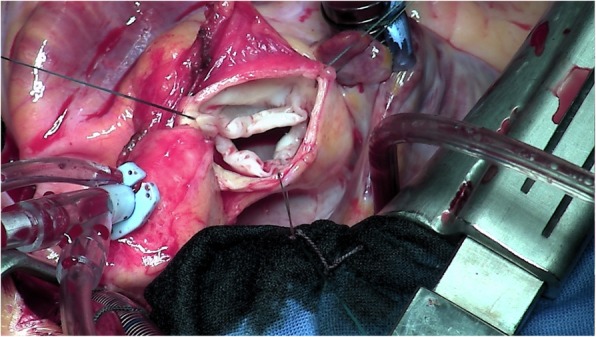
3 leaflet aortic valve showing fribromyxoid degeneration. The three leaflet are thick and partially retracted


Echocardiography showed severe aortic insufficiency, (regurgitant orifice and volume of 40mm^2^ and 95 ml respectively, video 1–3), of a tricuspid aortic valve with thick fibrous leaflets (Fig. 1), limited in their movement, and a dilated and hypertrophied left ventricle with conserved function. The patient underwent a modified Bentall operation with a 23 mm Medtronic freestyle porcine bioprosthesis. During operation, the root was found to be dilated (Z-score 3.2), and inspection of the aortic valve revealed non-calcified, retracted leaflets with white-coloured palpable indurated nodules localised to the free edges. A pathology investigation ruled out rheumatic disease and other identifiable causes, concluding that the valve expressed fibromyxoid degeneration. The post-surgical evolution was uneventful. She returned home after 7 days.

Additional file 1 **Video 1**. Short axis view: retracted thick aortic leaflets.



Additional file 2 **Video 2**. Long axis view: coaptation defect.



Additional file 3 **Video 3**. Long-axis view, Doppler: Large, central aortic regurgitation.


## Discussion

The interstitial deletion 2q31.2.q32.3 is well-known to be associated with developmental delay, behavioural troubles and dysmorphism. Here, we present an adult exhibiting these features with an additional finding of fibromyxoid degeneration of the aortic valve, which has never before been documented. Other causes of valvular degeneration were ruled out, in particular rheumatic heart disease was not present.

To our knowledge, no case reports concerning this particular interstitial deletion with early degeneration of the aortic valve exist. To date, only one report of an interstitial deletion in this region of chromosome 2 has described a congenital cardiac anomaly, however, it was a perimembranous ventricular defect discovered at birth and required operation at 10 weeks of age [2]. Furthermore, one family was described with a chromosome 2 deletion, involving latent transforming growth factor binding proteins which act in the TGF-β pathway, and presented an association with a thoracic aortic aneurysm. However, the deletion was located on the short arm of chromosome 2 [3]. The patient was also found having a severe scoliosis, requiring an operation. This disorder might also suggest an underlying connective tissue disease.

With respect to the operation, a repair was not possible as the leaflets were too damaged. We decided against a Ross operation because the aortic valve had been damaged by a fribromyxoid process, and we might have expected to see the same process within the pulmonary valve [4]. A complete valve reconstruction, such as an Ozaki procedure, could also have been an option, but there are currently not enough long-term studies in this area. Thus, the patient underwent a modified Bentall operation as described above.

## Conclusion

Interstitial deletion in chromosome 2q31.2q32.3 might be associated to a fibromyxoid degeneration of valvular leaflets generating regurgitation. Patients diagnosed with this mutation may require investigation to rule out a valvular disease.

Nevertheless, more cases are required before a clear association between this mutation and early degeneration of valvular leaflets could be confirmed.

## Data Availability

Not applicable.

## Abbreviation

TGF-β: Transforming Growth Factor beta

## References

[1] Prontera P, Bernardini L, Stangoni G, Capalbo A, Rogaia D, Ardisia C, Novelli A, Dallapiccola B, Donti E (2009). 2q31.2q32.3 deletion syndrome: report of an adult patient. Am J Med Genet A.

[2] Mc Cormack A, Taylor J, Gregersen N, George AM, Love DR (2013). Delineation of 2q32q35 deletion phenotypes: two apparent “proximal” and “distal” syndromes case rep genet.

[3] Quiñones-Pérez B, VanNoy GE, Towne MC, Shen Y, Singh MN, Agrawal PB, Smith SE (2018). Three-generation family with novel contiguous gene deletion on chromosome 2p22 associated with thoracic aortic aneurysm syndrome. Am J Med Genet A.

[4] Zakkar M, Mr BVD, Visan AC, Curtis S, Angelini G, Lansac E (2018). Stoica S Surgery for Young Adults With Aortic Valve Disease not Amenable to Repair. Front Surg.

